# Remimazolam‐Induced Paradoxical Reaction in a Bipolar Patient: A Case Report

**DOI:** 10.1002/ccr3.71624

**Published:** 2025-12-10

**Authors:** Zhifu Zhao, Li Liu, Xianjie Zhang

**Affiliations:** ^1^ Department of Anesthesia Deyang People's Hospital Deyang China

**Keywords:** bipolar disorder (BD), gamma‐aminobutyric acid (GABA), paradoxical reaction (PR), remimazolam

## Abstract

This first reported case of remimazolam‐induced paradoxical reaction (PR) in a bipolar disorder patient highlights that GABAergic dysfunction may heighten PR risk. Flumazenil effectively reversed the symptoms, underscoring the need for caution and preparedness when using benzodiazepines in this vulnerable population.

**Abbreviations**
BD: bipolar disorder
CNS: central nervous system
GABA: gamma‐aminobutyric acid
MOAA/S: alertness/sedation scale
PACU: postanesthesia care unit
PR: paradoxical reaction
SpO_2_
: oxygen saturation

## 1 Introduction

Benzodiazepines bind to the α subunits of gamma‐aminobutyric acid (GABA) receptors to enhance chloride ion influx, thereby exerting a sedative effect. However, PR may occur in some populations. Specifically, during or after a procedure, symptoms such as increased speech, emotional lability, excitement, excessive movement, and even hostility and anger may manifest [1]. The exact mechanism of PR remains unclear, and most cases are idiopathic. Nevertheless, evidence suggests that such reactions may be secondary to genetic associations, a history of alcohol abuse, or psychological disorders [2]. Remimazolam is a new type of short‐acting benzodiazepine, and it may be more likely to induce paradoxical reactions in populations at high risk of PR, such as patients with BD.

BD is a severe chronic mental illness characterized by cyclical changes in mood, namely the alternation between major depressive episodes and manic episodes [3]. The pathogenesis of bipolar disorder remains unclear. Previous studies have shown that dysfunction of the GABAergic neurotransmitter system may be involved [4], particularly GABAergic dysfunction in the hippocampus [5]. The patient in this case had comorbid bipolar disorder, with existing dysfunction of GABAergic neurotransmitters in the central nervous system (CNS). Consequently, the patient's sensitivity to sedative drugs underwent dynamic changes, which may have further amplified the risk of benzodiazepine‐induced paradoxical reactions.

By analyzing the clinical manifestations, mechanistic associations, and management experience of this case, this study aims to: (1) reveal the potential risk of remimazolam‐induced PR in special populations; (2) provide practical evidence for the optimization of perioperative sedation regimens in patients with mental illnesses. This study may fill the research gap regarding remimazolam‐induced paradoxical reactions in patients and facilitate clinical risk identification and decision‐making for drug selection.

## 2 Case History/Examination

A 13‐year‐old female patient (height 142 cm, weight 39 kg) was scheduled for elective gastroscopy under sedation due to a 3‐day history of abdominal pain, diarrhea, nausea, and vomiting. Her medical history was significant for bipolar disorder (BD), which had been diagnosed more than 8 months prior. She had been treated with a combination of fluoxetine hydrochloride (10 mg twice daily), lithium carbonate (125 mg twice daily), and sodium valproate (200 mg three times daily). After approximately 6 months of continuous pharmacotherapy, the patient and her family discontinued all medications without medical supervision, believing the condition had resolved. She had been off medication for over 1 month before the current admission and had returned to school without recurrence of mood symptoms; her Mood Disorder Questionnaire (MDQ) score was 0 at presentation.

Physical examination at admission was unremarkable. A genital examination was not performed. Upon arrival in the endoscopy suite at 09:05, her vital signs were stable: pulse rate 82 beats per min, respiratory rate 20 breaths per min, blood pressure 112/74 mmHg, oxygen saturation (SpO_2_) 97%, and body temperature 36.6°C. Preprocedural mask oxygenation was initiated promptly.

## 3 Differential Diagnosis, Investigations, and Treatment

Anesthesia was induced intravenously at 09:06 using remimazolam (10 mg), propofol (20 mg), and remifentanil (20 μg). Within 2 min, the patient achieved a Modified Observer's Assessment of Alertness/Sedation Scale (MOAA/S) score of 1, and the gastroscopy commenced. The procedure, which lasted approximately 4 min, was completed without incident or need for additional sedatives. Vital signs remained stable throughout, and the gastroscopic findings were consistent with chronic superficial gastritis. The patient was transferred to the postanesthesia care unit (PACU) immediately afterward.

At 5 min after PACU admission, the patient spontaneously opened her eyes, lifted her head, and sat upright, but exhibited an intermittently vacant and fixed gaze. Vital signs at this time were: pulse 76 bpm, respiratory rate 19 breaths/min, blood pressure 105/67 mmHg, SpO_2_ 99%, and temperature 36.6°C. One minute later (09:18), she abruptly developed acute psychomotor agitation characterized by high‐frequency, uncoordinated flexion‐extension movements of the lower limbs, tonic rolling of the trunk, and aimless, high‐frequency waving of both arms. She displayed marked panic, attempted to leave the bed, resisted physical contact, and demonstrated aggression toward medical staff and her mother, accompanied by screaming, verbal abuse, and intense hostility. Heart rate increased acutely from 75 bpm to 122 bpm; blood pressure and respiratory assessment were unobtainable during the episode.

The initial clinical impression was postoperative agitation or emergence delirium. An additional 8 mg of remimazolam was administered intravenously, leading to resedation after approximately 2 min of struggle. However, upon reawakening about 20 min later, the identical neuropsychiatric symptoms recurred. At this point, a paradoxical reaction (PR) to remimazolam was suspected, and flumazenil (0.4 mg IV) was administered at 09:44. Within 3 min, the patient's agitation resolved completely. She became calm, communicative, and oriented, with complete amnesia for the preceding events. During the episode of psychiatric symptoms, blood pressure, respiratory rate, and oxygen saturation could not be effectively monitored. Vital signs are presented in Table 1.

**TABLE 1:** Vital signs data.

Time	Blood pressure (mmHg)	Pulse rate (bests/min)	Respiration rate (times/min)	Temperature (°C)	SpO_2_ (%)
09:05	112/74	82	20	36.6	97
09:10	100/57	69	11	36.6	100
09:15	118/76	102	16	36.6	99
09:20	—	116	—	36.7	94
09:25	97/60	79	13	36.8	99
09:30	102/71	73	13	36.8	100
09:35	105/65	79	15	36.7	100
09:40	122/84	101	18	36.8	100
09:45	—	122	—	36.8	—
09:50	113/69	119	18	36.8	99
09:55	110/72	93	16	36.7	100
10:00	114/77	84	16	36.7	100
10:05	114/74	85	17	36.7	100
10:15	117/80	87	18	36.7	100
10:25	118/76	86	18	36.6	100
10:35	116/75	87	19	36.6	98
10:45	118/74	88	20	36.5	97

A psychiatry consultation was obtained, yielding a differential diagnosis that included postoperative delirium, a paradoxical drug reaction, or a manic episode. The patient was subsequently transferred to the psychiatric ward for inpatient observation. Comprehensive investigations—including liver and kidney function, thyroid function, coagulation profile, complete blood count, electrocardiogram, infectious disease screening, urinalysis, stool test, neurological examination, and brain MRI—revealed no abnormalities.

## 4 Conclusion and Results (Outcome and Follow‐Up)

**FIGURE 1:**
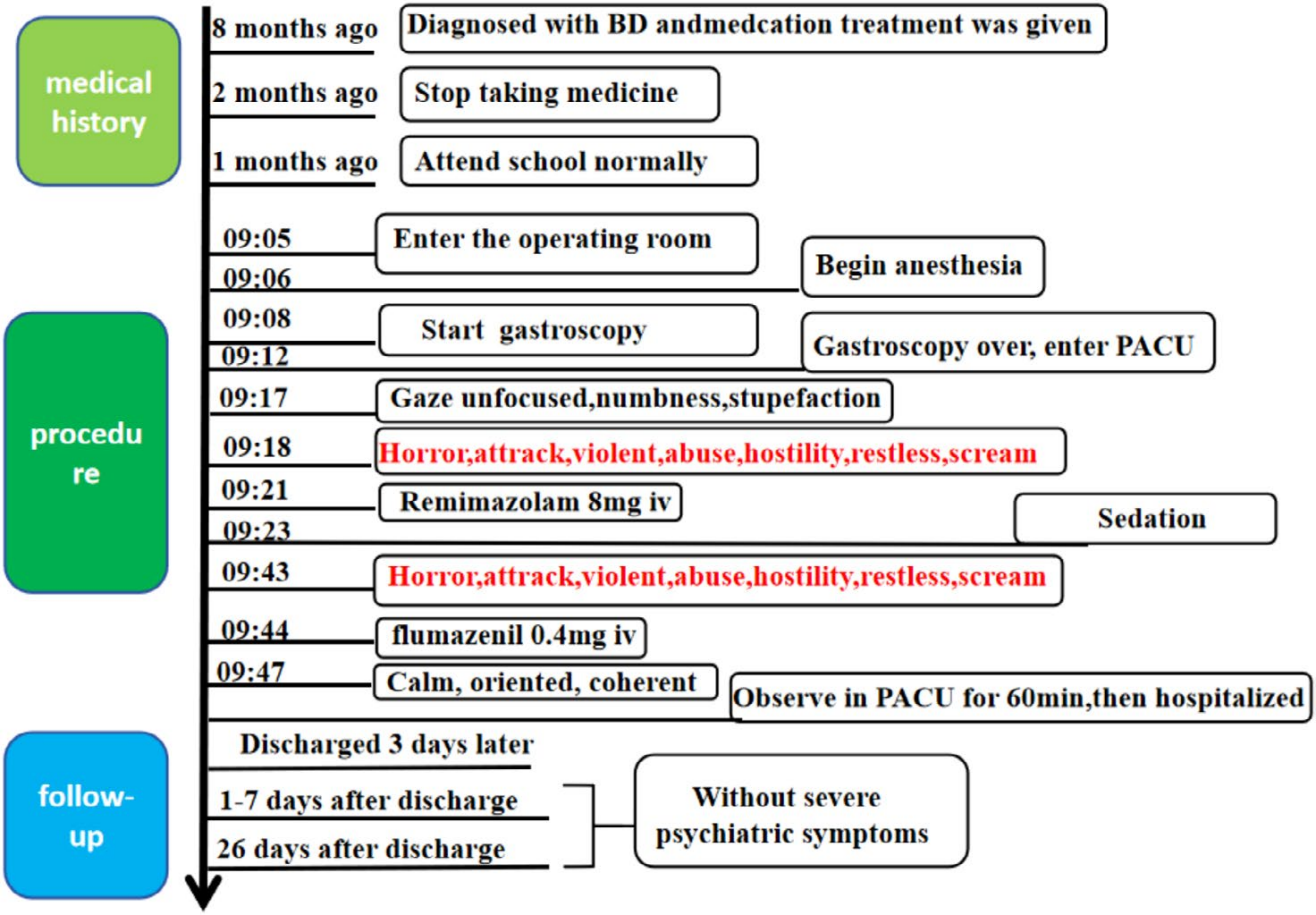
Timeline of episode.

The final diagnosis was a drug‐induced paradoxical reaction to remimazolam. This conclusion was supported by the transient nature of the symptoms (which did not meet the duration criteria for a manic episode) and the rapid, complete resolution following flumazenil antagonism. The patient received psychological support and was discharged on the third hospital day without resumption of psychotropic medications. Telephone follow‐up over 26 days postdischarge, including repeated MDQ assessments (score consistently 0), confirmed no recurrence of psychiatric symptoms, and the patient had successfully returned to normal school activities. The timeline of the episode is shown in Figure 1.

## 5 Discussion

We observed a case of PR during emergence in a patient with comorbid BD after sedation with remimazolam, which may be the first report of remimazolam‐induced PR. As pharmacokinetic and pharmacodynamic studies of remimazolam [6] have not mentioned PR as an adverse reaction, conversely, numerous studies have shown that remimazolam can be used to prevent and treat emergence delirium [7]. Therefore, when the patient first developed psychiatric symptoms, we did not consider PR, but instead suspected emergence agitation or delirium, and thus administered remimazolam again for sedation. After the second emergence, the patient still had obvious psychiatric symptoms, which led us to consider PR. Referring to cases where re‐administration of benzodiazepines was ineffective when PR occurred [8], we administered flumazenil for antagonism, and the patient's symptoms resolved quickly. A similar case was also reported by Jackson [9]. Hence, we highly suspect that the patient developed PR induced by remimazolam, a new‐type benzodiazepine. No recurrence of symptoms was observed during the subsequent 3‐day follow‐up, which ruled out the possibility of hypomanic or manic episodes and further confirmed the diagnosis of PR.

Reports of benzodiazepine‐induced PR are not uncommon [8, 10, 11]; PR can occur in all age groups, involving all drugs of this class and all administration routes [2]. Yi [12] also reported that the incidence of midazolam‐induced PR during endoscopic procedures ranges from 1% to 10%.

Cases of PR in patients with comorbid BD after benzodiazepine administration have also been reported occasionally. Gardos [13] reported a case of a 28‐year‐old female patient with comorbid depression who developed PR after taking diazepam, presenting with aggressive behavior toward relatives—highly similar to the current case. Strahan [14] also reported a similar study where a patient with comorbid BD developed PR manifesting as manic‐like episodes after using alprazolam. Additionally, PR induced by midazolam in patients with comorbid psychiatric disorders has been reported [15].

The mechanism of PR remains unclear, but some studies have identified potential predisposing risk factors, including young or advanced age, genetic susceptibility, alcohol dependence, and psychiatric illnesses and/or personality disorders [8]. PR may also be associated with mechanisms such as neuronal disinhibition due to excessive inhibition of the GABAergic system, hyperactivity of the limbic system, and individual differences in genetic sensitivity. The patient in this case had comorbid psychiatric illness (BD), which itself conferred a predisposing risk for PR. From the ligand‐receptor perspective, studies have confirmed that BD is associated with abnormal GABA (γ‐aminobutyric acid)‐ergic neurotransmission [16]. El‐Mallakh [17] found that patients with BD may also have abnormalities in intracellular chloride ion regulation mediated by GABA and its receptors. Further studies have revealed abnormal GABAergic neural function in the cingulate cortex of BD patient [18]. Given the patient's history of BD, we highly suspect that she may have had abnormalities in the GABAergic neuroendocrine system. When benzodiazepines act on the GABAergic system in an abnormal state, the potential risk of PR increases.

Of course, there are also some shortcomings in this case: (1) anesthesia used remifentanil and a small dose of propofol and the interaction between the two drugs or the chemical structure or pH change after co‐administration with remimazolam could not be excluded, which could induce psychiatric symptoms. (2) no genetic analysis was performed on the patient, so it is impossible to determine whether he has gene mutations in the GABAergic nervous system. (3) there was no check of blood drug concentration at the time of symptom onset.

## 6 Conclusion

This case represents the first reported instance of a paradoxical reaction (PR) induced by remimazolam in a patient with bipolar disorder (BD), highlighting critical clinical implications. The findings suggest that: (1) psychiatric patients, particularly those with BD and GABAergic dysfunction, may be at increased risk for PR when administered benzodiazepines; (2) remimazolam, as a novel benzodiazepine, can provoke PR in susceptible populations, warranting reassessment of its safety profile; and (3) flumazenil serves as an effective reversal agent for PR.

Clinical practice should incorporate thorough preoperative psychiatric evaluation, availability of reversal agents, and enhanced postoperative monitoring for high‐risk patients. Further research is needed to investigate the safety of sedative agents in psychiatric populations and identify predictive biomarkers for individualized risk assessment.

The patient's family expressed strong approval of the treatment approach and outcomes, particularly commending the medical team's professionalism and attentiveness.

## Author Contributions

**Zhifu Zhao:** conceptualization, data curation, methodology, project administration, writing – original draft, writing – review and editing. **Li Liu:** resources, writing – original draft, writing – review and editing, writing – review and editing. **Xianjie Zhang:** project administration, supervision, validation.

## Funding

The authors have nothing to report.

## Ethics Statement

This study was granted an ethical exemption by the Ethics Committee of Deyang People's Hospital. Written informed consent was obtained from the patient's legally authorized representative (father) for publication of this case report.

## Conflicts of Interest

The authors declare no conflicts of interest.

## Data Availability

The data supporting the findings of this study are available from the corresponding author upon reasonable request.
